# Citizens’ perspectives on healthy weight approaches in low SEP neighborhoods: a qualitative study from a systems perspective

**DOI:** 10.1186/s12889-024-19595-3

**Published:** 2024-08-07

**Authors:** Maud J. J. ter Bogt, Yentl Z. Te Riele, Piet G. C. Kooijman, Anita N. Heszler, Saskia van der Meer, Rob van Roon, Gerard R. M. Molleman, Maria van den Muijsenbergh, Gerdine A. J. Fransen, Kirsten E. Bevelander

**Affiliations:** 1 AMPHI Academic Collaborative Centre, Primary and Community Care, Radboud University Medical Centre, Nijmegen, EZ 6525 The Netherlands; 2 Municipal Health Service Gelderland-Zuid, Nijmegen, TV 6524 The Netherlands; 3 Primary and Community Care, Radboud University Medical Centre, Nijmegen, EZ 6525 The Netherlands; 4 Pharos, The Dutch Centre of Expertise on Health Disparities, Utrecht, LH 3507 The Netherlands

**Keywords:** Citizen science, Socioeconomic position, Neighborhoods, Healthy weight approach, Living environment, System science, Leverage points

## Abstract

**Background:**

The physical and the social environment are important predictors of healthy weight, especially in low socioeconomic position (SEP) neighborhoods. Many Dutch municipalities have implemented a healthy weight approach (HWA). Yet, there is room for improvement. This system science study examined what influences the utilization of HWA facilities and activities, and what aspects can help to achieve a desired systems change (also called leverage point themes (LPTs)) in the HWA system as perceived by citizens living in low SEP neighborhoods.

**Method:**

All research phases were performed with four citizens co-researchers. Forty-seven citizens living in low SEP neighborhoods were semi-structurally interviewed about the neighborhood HWA facilities and municipal HWA activities. A rapid coding qualitative analysis approach was applied per topic. The topics were citizens’ healthy living description, personal circumstances, and satisfaction with foot and cycle paths, sports facilities, playgrounds, green spaces, museums and theaters, community centers, churches, healthcare, school, food supplies, contact with neighborhood, unfamiliar and/or unused activities, familiar and used activities, unavailable but desired (lacking) activities, and reaching citizens.

**Results:**

The utilization of HWA facilities and activities was influenced by the overarching themes of social cohesion, familiarity, reaching citizens, maintenance, safety, physical accessibility, financial accessibility, social accessibility, fit with personal context, and fit with the neighborhood’s specific needs. Different overarching themes stood out across different facilities and activities. LPTs indicated the overarching themes needed in combination with one another for a specific activity or facility to increase utilization. For example, the LPT regarding foot and cycle paths was “accessible, safe, and maintained foot and cycle paths”. The LPTs regarding familiar and used activities were “customized activities; information provision (e.g., about possibilities to join without paying); social contact, meeting others, and everyone feels included”.

**Conclusion:**

Conducting inclusive qualitative research from a systems perspective among citizens living in low SEP neighborhoods has contributed valuable insights into their needs. This enables practical implementation of HWAs by providing a deeper understanding of the LPTs within the HWA system. LPTs can help HWA stakeholders to further develop current HWAs toward systems approaches. Future research could study the leverage points that may contribute to LPT implementation.

**Supplementary Information:**

The online version contains supplementary material available at 10.1186/s12889-024-19595-3.

## Background

Being overweight or obese is associated with reduced quality of life [1]. A person’s weight status and his/her lifestyle are explained by factors on multiple levels, with personal and social factors like income of major importance [2, 3]. In developed countries, people in a low socioeconomic position (SEP) are more likely to be obese [4, 5] and die sooner compared with those in a high SEP [6, 7]. Ample studies indicate the influence of the physical and the social environment on weight status, especially in lower SEP neighborhoods [5, 8, 9]. For example, citizens living in neighborhoods with limited availability of affordable, nutritious food, or with high fast-food restaurant density are positively associated with being obese [5, 10]. Further, adequate walkability, access to sidewalks and parks, high-quality recreational facilities within the neighborhood are negatively associated with being overweight [5, 8, 11–13]. Low SEP neighborhoods have fewer available and accessible facilities that promote regular physical activity [4] and healthy nutrition [8], suggesting that neighborhood facilities play a crucial role in health inequalities [3, 14, 15].

Municipalities arrange and influence the physical and the social environment by formulating and carrying out policies [16]. For example, in the Netherlands local prevention agreements are formulated in which municipalities make agreements with local partners about efforts to promote health and reduce health inequalities within their municipality [17]. Consequently, many municipalities in the Netherlands implemented a healthy weight approach (HWA) [16]. HWAs consist of all elements in a municipality that directly or indirectly influence the social and the physical environment regarding citizens’ healthy weight and determines the extent to which the living environment stimulates energy-balance-related behaviors among citizens. HWAs include both facilities (e.g., cycle paths, supermarkets, sport clubs) and activities (e.g., health-promoting interventions) organized by several stakeholders. To further enable citizens in a low SEP neighborhood to engage in healthy behaviors, HWAs should match the needs of low SEP citizens regarding a healthy lifestyle.

Recent literature indicates the importance of systems perspectives regarding HWAs and citizens’ lifestyle [18–20]. This systems perspective sees the HWA as a system that is non-linear, hard to control, and adaptable over time, where all elements are connected, thereby creating interdependency and feedback [18, 21]. According to Nobles et al. (2021), each system has four interconnected levels: (1) events: stakeholders’ outcomes and observable behaviors (e.g., system symptoms); (2) structures: the systems’ organization that causes events (e.g., relations, physical structures, information streams, patterns); (3) goals: the systems’ goals; and (4) beliefs: the systems’ deeply held attitudes, norms, and values [18]. Changes at deeper levels (i.e., goals and beliefs) are likely to change superficial levels too (i.e., events and structures). HWA activities and facilities are mainly linked to the events level, although underlying reasons about participation and use may be linked to deeper levels. Small changes in the system can improve the effectiveness of HWAs. A leverage point theme (LPT) describes aspects that can help to achieve a desired systems change when implemented [22, 23] and may occur at any of the four levels. For example, previous interviews with HWA professionals about HWAs indicated that positive messages about the HWA in citizens’ everyday language may be a LPT to encourage citizens’ motivation to utilize HWAs and health behaviors [22]. Insights into LPTs according to citizens can help to optimize HWA activities and facilities, as it may indicate what small changes are needed within HWAs.

The systems science literature addresses HWAs mostly from a professionals’ perspective [20, 22]. To our knowledge, the current systems science study is the first to investigate HWA approaches from an inclusive low-SEP-neighborhood citizens’ perspective, which is an important theoretical contribution to the current knowledge about HWAs and the physical and social environment. We adopted a citizen science approach in which citizens acted as co-researchers in our study. This approach contributes to insights about citizens’ specific needs, and these insights facilitate the practical implementation of changes in HWAs [24]. To enable stakeholders to strengthen the HWA, this study aimed to gain insights into what determines the benefits and utilization of HWA facilities and activities for citizens, by addressing the following research question: What social and physical environmental aspects influence the utilization of HWA facilities and activities, and what subsequent leverage points themes can be identified within the HWA system as perceived by citizens living in low SEP neighborhoods?

## Methods

### Design

A qualitative study with semi-structured interviews was performed. We adopted an inclusive research approach (i.e., citizen science), meaning that citizens were involved as co-researchers in the design, data collection and processing, interpretation, and dissemination of the results [25]. The Ethical Review board of Radboud University and Medical Center waived the need for a full review according to the Dutch Medical Research with Human Subjects Law (Wet Medisch-wetenschappelijk Onderzoek met mensen (WMO)) (registration number 2021–13172). We followed the ethical principles of the Declaration of Helsinki and GPDR regulations. All respondents received an information letter and gave written or oral informed consent.

### Study setting

Five municipalities in the Gelderland region of the Netherlands desired to strengthen their HWA and therefore started a four-year project where they organized learning communities. These five municipalities each had between approximately 19,000 and 41,000 citizens [26]. Two municipalities had between 200 and 500 citizens per km^2^, and three municipalities had between to 500 and 1000 citizens per km^2^ [27]. In each municipality, one low SEP neighborhood was selected based on two criteria: (1) the neighborhood had among the highest overweight rates according to the municipal health service’s public health monitor [28] and (2) the municipal health service’s health brokers characterized the neighborhood as low SEP. If more than one neighborhood met these criteria, the municipal health service’s health brokers identified the neighborhood with the greatest need for insights to strengthen the HWA.

### Co-researchers and respondents

At the start of the study, the co-researchers were recruited in the five municipalities (rather than the selected neighborhoods, as the neighborhoods were selected later in time) via advertisement posters in public spaces, such as supermarkets and community centers. Four co-researchers from two municipalities participated, as they contacted the researchers and wanted to become a co-researcher. The co-researchers were between 55 and 75 years old and 50% was male.

**Fig. 1:**
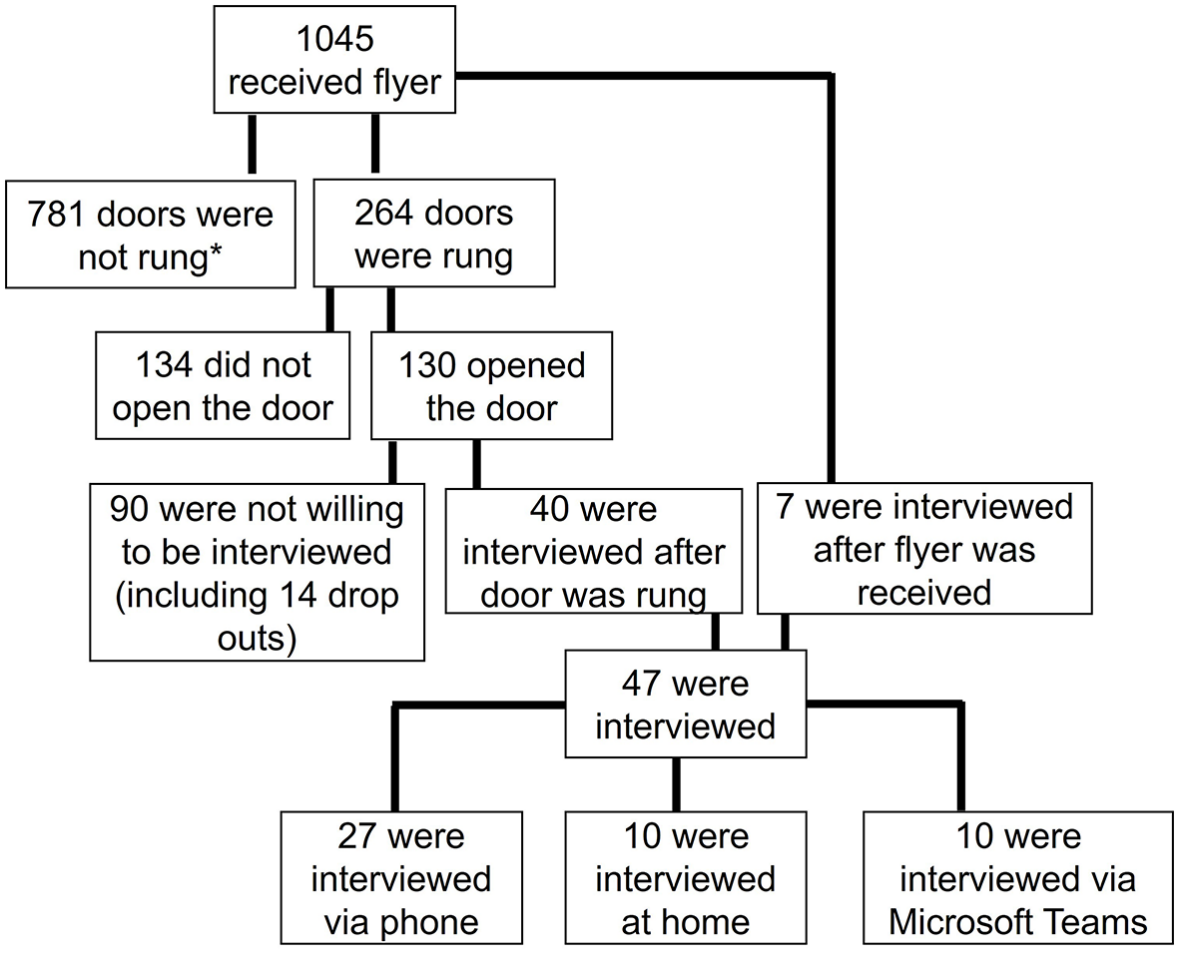
Recruitment diagram

The respondents were recruited in the five selected neighborhoods. We aimed to recruit 12 to 13 people per neighborhood, because this was expected to result in data saturation. Within every neighborhood, streets with a high population density in terms of flats or terraced houses and few green spaces were selected [e.g., based on 29, 30]. Observations in the neighborhood suggested that citizens living in these houses were most likely to live in a low SEP. To include a diverse group of citizens, the only inclusion criterion was that they were living in one of the selected areas. All 1045 selected houses received a flyer with study information and an interview invitation, and seven people signed up for an interview. Two to three weeks later, researchers together with the co-researchers went from door to door to recruit more respondents, resulting in 40 additional respondents (Fig. 1).

* These doors were not rung, because we reached the desired number of respondents.

The most frequent reasons given by the 90 people who did to not take part were not feeling like it (*n* = 29) and lack of time (*n* = 25). A few mentioned personal circumstances, such as not speaking the Dutch language (*n* = 5), health condition too bad (*n* = 3), not wanting to talk about health (*n* = 2), and not wanting to provide a reason (*n* = 12). Furthermore, some potential respondents could not be reached afterwards and thus dropped out, even though they were emailed or visited on three different days (*n* = 14). Our final sample consisted of 47 respondents. Most were overweight (68%), female (66%), and between 35 and 65 years old (51%), and many had a low level of education (45%) (see Table 1). In two municipalities, respondents were mainly female; in one municipality, they were mainly above the age of 65.

**Table 1:** Demographical characteristics of current study’s respondents

		Absolute number (%)
Municipality	1	10 (21%)
	2	9 (19%)
	3	9 (19%)
	4	11 (23%)
	5	8 (17%)
Body Mass Index^1^	18.5–25	14 (30%)
	> 25	32 (68%)
Sex	Male	16 (34%)
	Female	31 (66%)
Level of education^1^	Low	21 (45%)
	Medium	18 (38%)
	High	7 (15%)
Age (years)	18–34	4 (9%)
	35–65	24 (51%)
	65+	19 (40%)

^1^ One respondent did not want to provide height, weight, and level of education (*n* = 1 missing)

Citizens who agreed to take part were interviewed at a time that suited them via telephone, Microsoft Teams, or face-to-face at the respondents’ home, depending on their preference to enable inclusion of a diverse group of citizens. Researchers MB and YR (resp. PhD and MSc student, both female) conducted semi-structured interviews of approximately 30 min between March and May 2022, accompanied by the co-researchers when possible. A few respondents who did not feel comfortable about speaking in the Dutch language involved a family member/friend as interpreter. This seemed successful, as citizens who did not feel comfortable speaking the Dutch language also agreed to an interview.

### Study procedure

A semi-structured interview protocol was developed with the co-researchers. First, the facilities and activities were mapped. Facilities are visible places in the public domain that citizens can use, such as community centers or playgrounds. Activities are gatherings or programs organized by people, such as sports training or a consultation with a dietician. Desk research was performed by identifying all possible facilities in Dutch municipalities and neighborhoods that influence healthy behaviors from relevant literature [5, 8, 11–13]. Subsequently, they were clustered in the following facilities: cycle and foot paths; sports facilities; recreational areas such as playgrounds, green spaces, museums and theaters; facilities such as community centers, churches, healthcare, and schools; food suppliers such as fruit and vegetable sellers, restaurants, cafes, and snack bars; and facilities regarding neighborhood contact, such as contact moments with neighbors on the street [5, 8, 11–13]. Second, a focus group session with various involved HWA professionals (*n* = 5 per municipality) was organized in October 2021 to identify HWA activities per municipality. During this session, all activities organized in 2021 were mapped by the professionals, resulting in 11 to 19 activities per municipality. Third, pilot interviews were conducted among (co-)researchers and their relatives. Then, minor changes were made to question wordings, resulting in the final interview protocol.

The interview protocol started with introductory questions about respondents’ ideas and behaviors about healthy living (see Supplementary material 1), to prompt the participant to this topic. Next, per abovementioned facility, respondents were asked about satisfaction, use, accessibility, and positive and negative experiences. For all activities within the municipality, respondents were informed about the activity name and given a short description. Next, they were asked about their familiarity, use, and satisfaction per activity. In addition, they were asked what facilities were the most important, how activities could be promoted, and what activities were lacking. Lastly, they were asked their height, weight, and level of education, and the researcher observed their gender and age category. Participants were asked to think about their neighborhood when answering the questions.

### Inclusive research

All research phases were performed with four citizens who participated as co-researchers to increase study quality. In citizen science, five roles are distinguished that co-researchers can perform in different research phases, ranging from little involvement (informing) to a high level of involvement (control). To ensure that all co-researchers stayed involved throughout the entire study, we made sure that they could participate in a way that was comfortable for them. Table 2 describes the tasks and roles fulfilled by our co-researchers [31]. This process increased the accessibility and inclusivity of our research. For example, the co-researchers provided insights into what words were appropriate to use in the interview protocol, enabled the recruitment process (e.g., citizens identified more with them than the other researchers), and improved data interpretation (e.g., as they understood the local context).

**Table 2:** Involvement of co-researchers throughout the research phases [inspired by 31]

	Role played by co-researchers				
Research phase	Informing	Consulting	Involvement	Collaboration	Control
Design research question			Brainstormed and formulated research question together with researchers		
Interview protocol				Brainstormed together with researchers, afterwards a draft version was created by the researchers, which was adapted in collaboration with co-researchers	
Respondent recruitment	Neighbor-hoods and streets were chosen by researchers, health brokers, and policymakers			Recruitment strategy decided upon together with researchers; the recruitment flyer was made by a co-researcher and a researcher; respondents were recruited together with researchers	
Data collection			Researchers performed interviews; co-researchers were present as second interviewer when co-researchers wanted to (21% of interviews), i.e., availability and co-researchers preferences.		
Data analysis				Researchers transcribed most interviews; co-researchers transcribed the number of interviews they wanted to (these were afterwards checked by the researchers)	All summaries were interpreted with co-researchers; overarching themes, links to the Action Scales Model, and LPTs were identified together
Designing end product				A presentation for the learning communities was designed and executed together	The flyer for respondents was mainly made by the co-researchers

### Data analyses

Interviews were audio recorded and transcribed ad verbatim. We used rapid coding qualitative analysis to structure the large number of interview transcripts. This process consisted of the following five steps [inspired by 32–34]. First, one of the co-authors (YR) summarized five transcripts and organized the content into topics^1^ aligned with the interview protocol (see Study procedure), including the facilities and activities and adding one more topic “other” (including personal circumstances). Summaries stayed close to respondents’ words. The summaries were checked and discussed by two researchers (YR and a research assistant) until consensus was reached. Next, a researcher summarized the remaining interviews per topic (YR), and unclarities were discussed with the main author (MB). Second, based on the summaries per topic per respondent, one summary per topic was created for all respondents within one municipality (YR). These were randomly checked for completeness by the main author (MB). Third, researchers YR and MB interpreted the summaries per topic per municipality together with the co-researchers during a group discussion, until data saturation was reached. For example, the co-researchers explained the respondents’ answers regarding their feeling that healthy living includes nutrition and exercise as well as mental aspects such as fun and relaxing. Fourth, the summaries per topic per municipality were summarized in one overall summary per topic by the main author (MB). In addition, the activities were categorized into “unfamiliar and/or unused activities” and “familiar and used activities”. One co-researcher checked the overall summaries per topic for completeness. Fifth, the main author (MB) interpreted the overall summaries per topic together with the co-researchers during a group discussion.

Based on these five steps, overarching themes across the topics were identified and discussed by the co-researchers and main author (MB), and afterwards discussed among co-authors (MB, KB, GF) until consensus was reached. This resulted in 10 overarching themes. A topic can be associated with multiple overarching themes. For example, the foot and cycle paths topic was associated with the overarching themes maintenance, safety, and physical accessibility. As a subsequent and final step in our analysis, the main author (MB) and the co-researchers linked the topics to (1) the four levels of the Action Scales Model by coding the overall summaries on the four levels (as further elaborated in Supplementary material 2) and (2) identified LPTs in a group discussion with co-researchers guided by the question: What should happen for respondents to use [topic] much more?, followed by a discussion between three authors (MB, KB, GF) until consensus was reached [18]. Whereas overarching themes indicate one specific aspect that determines the extent to which HWA facilities and activities were used, LPTs indicate what is needed in combination with one another to use an activity or facility much more. The overarching themes, LPTs, and links to the four levels of the Action Scales Model are described in the results section.

## Results

Data analyses resulted in 10 overarching themes: familiarity, reaching citizens, maintenance, safety, physical accessibility, financial accessibility, social accessibility, fit with personal context, fit with the neighborhood’s specific needs, and social cohesion. These overarching themes are explained below. Every overarching theme only related to some activities and/or facilities, but not others. It depends on the activity and facility which of the overarching themes are of influence and relate to what system levels, as illustrated in Table 3.

**Table 3:** Overview of topics, overarching themes, underlying LPT and the main corresponding system levels

Topic	Overarching theme^1^	LPT	ASM level^2^
Healthy living description	Fit with personal context	Facilities and activities should align with the way in which citizens describe healthy living (e.g., healthy nutrition, feeling safe, trying to relax)	EGB
Personal circum-stances	Fit with personal context	Facilities and activities should align with personal circumstances (e.g., loneliness, working a lot, tight budget, being chronically ill)	ESGB
**Facilities**			
Foot and cycle paths	Maintenance, Safety, Physical accessibility	Foot and cycle paths should be physically accessible, safe, and well maintained	ESB
Sports facilities	Familiarity, Physical accessibility, Financial accessibility, Fit with the neighborhood’s specific needs	Local sports facilities should be accessible in terms of costs and physical distance; local sports facilities should consist of a bigger offer of accessible, public sports facilities	ESB
Playgrounds	Familiarity, Maintenance, Safety, Physical accessibility, Fit with the neighborhood’s specific needs	Challenging playgrounds should be available for all age groups and be well maintained, safe, and physically accessible	ESB
Green spaces	Familiarity, Maintenance, Safety, Physical accessibility	Green spaces should be well maintained and accessible for disabled people; safety at the dike	ESB
Museums and theaters	Familiarity, Financial accessibility	Not applicable, because respondents did not mention any aspects that should be present to use the facility much more	ES
Community center	Familiarity, Maintenance, Social accessibility, Fit with the neighborhood’s specific needs	Everyone feels welcome at the community center (e.g., activity offer for more diverse age categories, open community centers where non-Reformed citizens also feel welcome)	ESGB
Churches	Familiarity, Social accessibility	Everyone feels solidarity/togetherness	ESB
Healthcare	Familiarity, Physical accessibility, Social accessibility	Healthcare should include a findable place with information about local healthcare; GP should be present in the neighborhood	ESB
School	Reaching citizens, Social accessibility, Fit with the neighborhood’s specific needs	Not applicable, because respondents did not mention any aspects that should be present to use the facility much more	ESB
Food supplies	Physical accessibility, Financial accessibility	Food supplies should be affordable, healthy, and available in the neighborhood; healthy living should be promoted; unhealthy food should be limitedly available	ESGB
Contact with neighborhood	Social cohesion	Everyone experiences good contact with the neighborhood (e.g., talking to one another, helping one another, less nuisance)	ESGB
**Activities**			
Unfamiliar and/or unused	Familiarity, Fit with the neighborhood’s specific needs	Activities should be promoted among citizens and citizens should be convinced about the importance of activity participation; activities should be accessible (e.g., affordable)	ESGB
Familiar and used	Maintenance, Physical accessibility, Financial accessibility, Fit with the neighborhood’s specific needs	Activities should be customized; activities should contain information provision (e.g., about possibilities to join without paying); activities should include social contact, meeting others, and everyone feels included	ESGB
Activities lacking	Fit with the neighborhood’s specific needs	Activities should relate to sports and/or social contact, and be free/cheap; activities should be customized to personal choice and obligation	ESGB
Reaching citizens	Reaching citizens	To reach citizens, places should be used where citizens frequently are, personal attention, careful listening and responding to this	SB

^1^ 1 = familiarity; 2 = reaching citizens; 3 = maintenance; 4 = safety; 5 = physical accessibility; 6 = financial accessibility; 7 = social accessibility; 8 = fit with personal context; 9 = fit with the neighborhood’s specific needs; 10 = social cohesion

^2^ E = events; S = structures; G = goals, B = beliefs

### Familiarity

A precondition for using facilities and activities was the respondents’ familiarity with them. Most respondents indicated that they were familiar with facilities such as green spaces, healthcare (e.g., general practitioner), sports facilities, playgrounds, community centers, churches, and schools in their municipality. However, they made use primarily of green spaces and healthcare facilities. The facilities and activities that were least familiar to respondents and often not used were museums and theaters. In general, respondents were not familiar with many HWA activities. The most familiar facilities and activities were consultations at the youth healthcare center and sports activities, because they used them themselves or knew other people using them. Why facilities and activities were used or not was influenced by the overarching themes explained below.

### Reaching citizens

The promotion of HWA activities among citizens was found important because many activities were unfamiliar to the respondents. Many respondents indicated that they wanted to be reached by communication and information about activities via printed information such as papers, letters, or folders. Some respondents wanted to be informed via social media or personal contact.

Respondents indicated that they frequently visited places close to home that could be used to reach and inform them about activities, such as the supermarket, school, library, or the village center. Further, some respondents mentioned nature spots (e.g., floodplain, dike), places near their home (e.g., street, passing by front door), social contacts (e.g., at neighbors), leisure spots (e.g., associations, swimming pool), meeting places (e.g., terrace), or medical spots (e.g., GP, pharmacy).

This shows that reaching citizens remains difficult, as everyone desired something different. In general, the respondents desired mainly personal attention, careful listening, and actions upon this information:

“Going by people. […] I think that’s a good thing, because then people actually do listen.” (Municipality 3_Citizen 5, age range 65 years or older).

### Maintenance

Respondents indicated that adequate maintenance was an important aspect for the use of facilities such as foot and cycle paths, playgrounds, green spaces, and community centers. For example, if activities took place in an outdated facility, this was perceived as a barrier to participation. The maintenance of big green spaces outside the neighborhood was perceived adequate in most municipalities. Respondents mentioned that maintenance of green spaces in the neighborhood was important (e.g., removal of weeds, cutting hedges, cleaning dog poop), because it facilitated usage (e.g., use with wheelchair). Limited maintenance of foot and cycle paths was perceived as a barrier mainly by older adults because they are afraid of falling:

“Then I walk on the sidewalk. But then I walk with the walking frame, but it [the sidewalk] is so crooked as I don’t know what. You really have to watch out. […] I sometimes go out with someone, and then I walk on the sidewalk. Then there’s someone there, but I wouldn’t do it [walk on the sidewalk] alone.” (Municipality 2_ Citizen 12, age range 65 years or older).

### Safety

Safety was perceived as important regarding foot and cycle paths, green spaces, and playgrounds. Lights, traffic calming devices, and sufficient space to pass one another were indicated as contributing to the safety of foot and cycle paths. Respondents mentioned that they felt less safe in situations where it was relatively narrow for all the traffic to pass, for example, around dikes. This also influenced respondents’ nature experience. For children, most playgrounds were perceived as safe thanks to fences surrounding the playgrounds, but remote playgrounds or playgrounds along the water were perceived as unsafe, thereby influencing their usage:

“Safety, of course, because there is also a large fence around [the playground].” (Municipality 4_Citizen 6, age range 65 years or older).

“That [playground] is a bit hidden, that you think; well, it’s fine that it’s there, but as a mother you have to come along. It’s not a place where you leave your kids alone, and that eh, that’s also that you just do not do it [let my kids play at the playground]. They [the playgrounds] could be more centrally located in the neighborhood, in my opinion.” (Municipality 5_Citizen 12, age range 18–35 years old).

### Physical accessibility

Facilities and activities that are accessible locally in terms of physical distance were perceived important regarding foot and cycle paths, sports facilities, playgrounds, green spaces, healthcare, food supply, and activities. For example, playgrounds were scattered around the neighborhood and therefore nearby for everyone, making them feel accessible and thereby facilitating playground usage. Moreover, when healthcare facilities were clustered, meaning that the GP, physiotherapist, dietician, and pharmacy were situated close to one another, this increased the perceived accessibility. Still, some citizens had to find a GP outside their neighborhood, which was not appreciated:

“Of course, we don’t have the general practitioner in [name neighborhood]. So, then you have to go to [another village]. And that is of course a disadvantage. Especially when you get older.” (Municipality 3_Citizen 11, age range 65 years or older).

Regarding food supply, limited accessibility of greengrocers or supermarkets was not appreciated:

“The greengrocer, I mean, it makes a difference with the supermarket. And that too is, so to say, at a neutral place […]. You have for example, further down […] vegetable shops […], but then you have to go for a long way [by] bike or by car.” (Municipality 4_Citizen 12, age range 18–35 years old).

Some respondents mentioned the too prominent accessibility of multiple snack bars that offered unhealthy food:

“I don’t think there is […] promoting so much about healthy living or anything. It is full of snack bars here. I know plenty of places where I can get very unhealthy food. […] You are not pushed to live a healthy life, in terms of nutrition.” (Municipality 1_Citizen 1, age range 35–65 years old).

When an activity took place outside the neighborhood, this was perceived as a barrier to participation:

“Only that is again another village further, so then you are always dependent on the car.” (Municipality 4_Citizen 12, age range 18–35 years old).

### Financial accessibility

Respondents desired affordable sports facilities, food supplies, and activities. Multiple respondents indicated that their financial situation precluded them from engaging in activities.

Further, healthy nutrition was perceived as expensive, which was not appreciated:

“The healthier you want to live, the more expensive it is.” (Municipality 4_Citizen 10, age range 35–65 years old).

“That is the problem everywhere. If you don’t have money, then you cannot use [it].” (Municipality 5_Citizen 3, age range 35–65 years old).

Moreover, free public outside sports facilities were available in two municipalities and were lacking in other municipalities:

“If you don’t have access to an association or something like that or are not financially adequate, I don’t see many public sports places like a park or eh, eh, a jogging track so that you, eh without costs, can exercise.” (Municipality 1_Citizen 10, age range 35–65 years old).

### Social accessibility

Social contact, meeting others, and the feeling that everyone is welcome during an activity was perceived as a facilitator for participating in various activities and facilities, whereas activity and facility participation was limited when respondents experienced the feeling of being left out, for instance because of cliques of people:

“There is only a very limited group of people, so to speak, always and always the same people. So, a lot of other people just never get there. Whereas those facilities are actually for general for everyone.” (Municipality 5_ Citizen1, age range 35–65 years old).

Social accessibility also determined the extent to which community centers, churches, healthcare, and schools in the municipality were used and appreciated. For example, respondents in most municipalities indicated that the community center was perceived as a meeting place and accessible for everyone regarding activities or volunteering, whereas some respondents felt left out because needs of their personal circumstances were not met. One respondent felt socially isolated because of his chronic illness and occupational disability:

“I can’t fulfill an agenda […]. I don’t know how I’ll wake up tomorrow and how I’ll walk. So yes, but then they can’t count on me, so I’m left out [from volunteering]. I have already experienced that in other clubs.” (Municipality 5_Citizen 4, age range 35–65 years old).

Furthermore, social contact was influenced by the role of religion in several facilities, such as schools and community centers. For example, in some municipalities, there were schools and community centers that were perceived as available only for people belonging to a particular religion:

“You have a public school […] and the Dutch Reformed school […] everyone can go there too. And then you have again, two Reformed schools, there you really have to be, uhm, from that church, so to speak. Otherwise, you will be refused.” (Municipality 4_Citizen 3, age range 35–65 years old).

“You do have certain neighborhood buildings, but that is also very Reformed, so that is actually more a bit from them [the Reformed community] so to speak. So as an outsider you don’t, don’t get in there […]. So, it would also be nice if they would open a community center for those who are not Reformed.” (Municipality 4_Citizen 12, age range 18–35 years old).

In addition, respondents in most municipalities indicated that the church was perceived as accessible for everyone, e.g., because they organized activities (e.g., drinking coffee with others, gatherings). Only in one municipality was religion perceived as determinative and as a source of togetherness and exclusion:

“What I always find, uhm, very admirable, is that you often see in a church community that there is solidarity. Uhm, that people think less individually, but more about one another […]. But in terms of religion, it is such that […] a great deal of disagreement has come. That there were even quarrels within families and that separate church meetings were arranged.” (Municipality 4_Citizen 10, age range 35–65 years old).

“[If you] don’t go to church, yes, they [the church community] do look down on you. […] And then I think, yes, you should also respect us. […] Look, now we have King’s Day tomorrow and then they will have a separate party, do you understand? […] [Then I think] yes guys, don’t do that and just make it a village party, right?” (Municipality 4_Citizen 3, age range 35–65 years old).

Moreover, in most municipalities, respondents were positive about the healthcare received, for example, because healthcare professionals were largely involved with patients. A few respondents, however, felt rejected. For example, in one municipality, respondents were not positive about their GP, because they clashed.

### Fit with personal context

Respondents explained that it was important for facilities and activities to align with their personal circumstances. Personal circumstances included feelings of loneliness as a result of the loss of significant others (e.g., passing away or being a refugee from Syria), not speaking the Dutch language, working a lot, having a tight budget, or being chronically ill and therefore feeling rejected and isolated. Therefore, they appreciated activities and facilities that facilitated personal contact. Yet, these personal circumstances often resulted in their not being able to attend facilities and activities in the municipality:

“I am completely declared unfit. […] a lot of things are physical. Yes, those are not possible for me. And that’s shitty. If only that were true, because that would also take me out of my isolation, because then I have something to go to again.” (Municipality 5_Citizen 4, age range 35–65 years old).

“Now, I know only, done with language, I want to [first] speak good Dutch, and [then] also go [to] activity or go to good work.” (Municipality 4_Citizen 13, age range 35–65 years old).

“I have worked a lot. And then I am glad I can rest at home at night.” (Municipality 1_Citizen 3, age range 35–65 years old))

Respondents felt that facilities and activities should align with their definition of healthy living. They described healthy living in terms of having a healthy diet and exercising, as well as broader topics, such as being happy, having less stress, clean air, sufficient sleep, feeling safe, and using aid related to healthcare. Therefore, activities and facilities that aligned with their definition were often appreciated, whereas other activities and facilities should be more about these topics.

On activity level, respondents indicated that matching activities with their needs and convincing them about their importance facilitated activity participation. For example, some respondents mentioned reasons not to participate in activities, including not feeling the urgency, not wanting to be tied down, having no time, not liking the activity, doing comparable activities themselves, or not feeling part of the target group. For example, both the Healthy School initiative’s healthy lifestyle stimulation and positive tailored guidelines from the dietician and child consultation office were appreciated by many respondents. Yet, these initiatives also felt commercialized and raised feelings about being pushed and told what to (not) do, which was not appreciated by some. Still, they liked that they were stimulated to go to the gym. Overall, some respondents felt that professionals could not customize advice to the individual situation. Therefore, activity customization was important:

“It [rules of the Healthy School] is forced upon you.” (Municipality 4_Citizen 10, age range 35–65 years old).

“A bit of a crazy situation […]. They [my children] are absolutely not allowed to bring bread or crackers to school.” (Municipality 2_Citizen 7, age range 65 years or older).

“I did not enjoy going there [the consultation office] […]. Where you will be rapped on the knuckles again, wouldn’t you just do it according to the book?” (Municipality 3_Citizen 3, age range 18–35 years old).

Although respondents indicated that they did not desire any activities that were lacking in the HWA, some mentioned that they would have liked activities about meeting people, exercising, a walking group, and an information evening about healthy nutrition. Altogether, the respondents desired more activities related to sports and/or social contact that were free or cheap. Some respondents desired accessible and non-committal information from an exercise coach or dietitian, whereas others desired an obligated level of intensity (e.g., a phone call if you did not come). Therefore, customization in the extent of personal choice and obligation was important.

### Fit with the neighborhood’s specific needs

Various needs of the neighborhood were mentioned. For example, more challenging playgrounds and playgrounds for older youth were lacking. In addition, a variety in activities within community centers were desired, as few activities or activities only for the elderly were organized. Regarding sports facilities, shortcomings were experienced, e.g., limited sports variety, limited time slots, being too far away, sports offer stopped because of too few children, or too few volunteers. Therefore, more sports offerings in the neighborhood were desired:

“[There is] especially soccer and I personally find that very unfortunate. I’m not much of a soccer person so that’s why.” (Municipality 2_Citizen 9, age range 35–65 years old).

“They are both playing soccer […]. It is a disadvantage of having two villages mixed together. They then have to train here in [name neighborhood] for six months. And the other half year in [name of other village].” (Municipality 3_Citizen 5, age range 65 years or older).

### Social cohesion

Social cohesion within the neighborhood was perceived as important regarding healthy living and the HWA, as it relates to the overall community feeling within the neighborhood. For example, contact with neighbors was perceived as important and took place mainly close to home, e.g., in front of the house, or in the street. In all municipalities, contact with the neighborhood was perceived good, as neighbors had a chat, greeted one another, and helped one another when needed:

“For example, my boyfriend always goes with him [man in the neighborhood], say cycling, so they go race cycling together.” (Municipality 5_Citizen 12, age range 18–35 years old).

“We [the neighbor and I] regularly go for a cup [of coffee] or something. And if I call the neighbor here, I have such a computer, but I can’t use it at all. And the neighbor, I only have to knock and say there is this or that, well, then she is there and she helps me too.” (Municipality_2 Citizen_2, age range 65 years or older).

Still, respondents mentioned disadvantages of living in a village, such as gossip and social control. In some neighborhoods, respondents also experienced little contact and nuisance, as a result of clashes and arguments with neighbors, noise, or drugs. Furthermore, some respondents perceived making contact with the neighborhood as hard. For example, contact with the neighbors was perceived as less intense among new people who moved from another municipality to the neighborhood compared with the older generation who lived in the neighborhood for years:

“It’s really the older ones, so to speak, the older generation they may be social and involved, but the new ones, the younger ones from the outside are not. […] They [the new ones] can’t even say hello properly anymore. […] With neighborhood day […] the regulars come but you don’t see the new ones.” (Municipality 4_Citizen 5, age range 35–65 years old).

### Leverage point themes

A combination of the overarching themes were often perceived as important within a topic. For example, regarding the playgrounds topic, the five overarching themes familiarity, maintenance, safety, physical accessibility, and fit with the neighborhood’s specific needs were important in combination with one another (Table 3). Yet, regarding the personal circumstances topic, only the overarching theme fit with personal context stood out.

Likewise, LPTs were identified per topic, and LPTs were related to combinations of overarching themes. For example, the LPT regarding playgrounds was “challenging playgrounds for all age groups that are well maintained, safe, and physically accessible” (Table 3). The LPTs regarding familiar and used activities were “customized activities and information provision (e.g., about possibilities to join without paying); social contact, meeting others, and everyone feels included”. For museums and theaters and schools no LPTs were identified, because respondents did not mention any aspects that should be present to use the facility much more. Table 3 presents per topic an overview of the corresponding overarching themes, LPTs, and systems levels.

All four systems levels were identified in the analysis. Events, structures, and beliefs were the most common, whereas goals were absent for most topics. For example, the personal circumstances topic revealed that some respondents were not able to participate in facilities and activities (events) because their personal circumstances (structures) were difficult, and this caused feelings of rejection and isolation (beliefs), even though some respondents aimed to participate (goals). Most topics covered at least three of the four systems levels.

## Discussion

The current study was the first to examine how the HWA was experienced by citizens from five Dutch low SEP neighborhoods from a systems science perspective. In general, the HWA activities and facilities were more likely to be used if citizens from low socioeconomic backgrounds were familiar with them and feel socially included. The results indicated that multiple factors must be combined to stimulate the use of HWA activities or facilities among citizens in low SEP neighborhoods. This combination is united in an LPT. Different HWA activities and facilities serve different citizens’ needs, requiring different factors. For example, foot and cycle paths were more likely to be used when they were perceived as well maintained, safe, and physically accessible. On the other hand, sports facilities were more likely to be used if they were physically accessible, financially accessible, fitted with the neighborhood’s needs, and were familiar to citizens. HWA stakeholders are recommended to implement these LPTs per HWA activity and facility by taking small and specific steps [18, 22]. For a few HWA facilities, however, no LPTs were found as citizens did not mention aspects that would stimulate their usage. For example, citizen perspectives did not result in an LPT regarding schools. Yet, other studies have indicated that professionals perceive health policies at schools as a method to change citizens’ healthy behavior beliefs [35]. Moreover, this research indicates that the HWA themes are interpreted broader by citizens than researchers usually do. This indicates that professionals’ and citizens’ perspectives about HWAs differ, and both perspectives need to be taken into account to strengthen HWAs.

Social inclusion and social cohesion emerged as important themes within the HWA according to citizens; this emphasizes the importance of the social aspect. For example, contact with neighbors was perceived as important regarding healthy living and the HWA. Moreover, the perception of community centers as a meeting place is in line with previous research that referred to local, public social places such as community centers as third most important place of social interaction after home and workplace [36]. Feeling socially excluded from HWA facilities and activities was linked to religion, personal circumstances, or believing that most activities were organized for another target group. Previous research also suggested that social exclusion was more apparent among people in a low SEP than people not in a low SEP [37]; this suggests that low SEP citizens are more likely to feel excluded. Previous research also suggested that local-level social inclusion or exclusion differs between neighborhoods and may have an effect on health [38]. It is thus important to ensure that everyone feels welcome at HWA activities and facilities such as community centers (LPT), for instance by organizing activities and facilities among different age and culture groups by developing HWA activities and facilities together with the target group.

Further, familiarity with HWA facilities and activities was identified as a significant factor in the HWA. Even though a broad activity and facility offer is available within the municipalities, citizens have a limited awareness of this offer. This is supported by previous studies among professionals and citizens regarding the HWAs in the same municipalities [22, 39], as they also indicated that citizens were limitedly reached. To overcome this, professionals should focus on reaching low SEP citizens by personal attention at places where citizens frequently are (LPT), for example by using familiar locations, creating informal conversations, using understandable language, creating an environment where language is less important, involving community members, and relying on easy-to-use information methods to limit digital inequality [40–43]. Moreover, the overarching theme, financial accessibility, was important among various HWA activities and facilities. In line with this finding, previous research in the same region has indicated that low SEP citizens who aim to improve their health prioritized solutions to reduce financial stress, rather than healthy eating and physical activity [39]. Therefore, HWA stakeholders are encouraged to make HWA activities, sports facilities, and healthy food offerings more financially accessible (LPT), for instance by enabling cheap usage of these facilities, national income policies, or distributing the municipality’s financial allowances to citizens who cannot afford these activities.

In the current study, the Action Scales Model provided levels to portray how a complex HWA system operates [18] and to discover LPTs from a citizens’ perspective that may strengthen HWA utilization when implemented. In our study, the results revealed LPTs across all systems levels. Some topics and LPTs were linked to deeper systems levels (goals and beliefs) than others. HWA professionals are recommended to implement LPTs within their sphere of influence and to prioritize implementation of LPTs relating to deeper systems levels (such as personal circumstances rather than museums and theaters), as these are more likely to have impact [18]. Still, LPTs regarding events and structures are also important, as they may function as boundary conditions in a HWA system [22]. For example, regarding the personal circumstances topic, some respondents did not participate in facilities or activities in the municipalities (event) because of a mismatch with personal circumstances, such as being chronically ill (structure). Still, the respondents wanted to participate in social HWA activities (goal), because that would reduce their isolation (belief). Implementing LPTs may support the implementation of systems thinking within HWAs and may increase HWA utilization. Promising future research could study what specific leverage points may contribute to the implementation of the LPTs as discovered in the current study. For instance, a thorough systems science study (e.g., causal loop diagrams) among citizens may yield insights into underlying feedback loops to understand mechanisms and potential unintended consequences. Yet, to enable citizen science and community engagement in such a study, adapted group model building methods may be required, such as by using participatory methods (e.g., photo voice) and working together with citizens as co-researchers.

Altogether, in the studied HWAs, there seems to be a mismatch between the existing HWAs and low SEP citizens’ personal circumstances. Matching the activities and facilities with citizens’ personal circumstances is thus regarded as an LPT, for instance giving a lift to residents who cannot go to the activity themselves. This finding is in line with previous research that suggested limited activity participation resulting from time pressure from work or domestic life [44], low income [45], and different needs among chronically ill and disabled citizens [46]. To further adapt HWA activities and facilities to low SEP citizens’ needs and reduce health inequalities, professionals could, on the one hand, invest in customizing HWAs to citizens’ needs – for example, by implementing buddy systems, customizing activities and facilities, or co-developing activities and facilities with citizens. On the other hand, previous research has demonstrated the importance of environmental solutions [8, 9], such as a healthy living environment to influence social norms and make the healthy option the easier option. Such a community-wide approach cannot be customized to all individuals in the neighborhood. Therefore, actions that originate from solutions created both by professionals (top-down) and by citizens (bottom-up) should be intensified. To combine both approaches, HWA stakeholders are recommended to engage citizens via citizens’ front door [40, 41, 47], but funding streams and organizational legitimacy may make some stakeholders perceive this as difficult [48]. This suggests that changes to the HWA system might be needed to intensify the adaptation of HWA facilities and activities to citizens’ needs based on both top-down and bottom-up approaches.

### Strengths and limitations

A main study strength is the collaboration with co-researchers throughout the entire research process and the participation of citizens with a diversity of backgrounds, which enabled insights to be gained into LPTs for a low SEP citizens. Still, we recommend future research to collaborate with co-researchers who are as comparable as possible to the target group, which would refer to citizens living in the selected low SEP neighborhoods in the current study. Moreover, applying the Action Scales Model with co-researchers enabled the results to be arranged into LPTs. A study limitation is that we examined citizens’ perceptions, meaning that we described perceived LPTs but are unsure as to whether there are other LPTs within the system. Another limitation is that women above the age of 35 years are overrepresented, even though the respondents’ demographic characteristics were diverse. Lastly, HWAs of five municipalities within the Gelderland region (the Netherlands) were studied, but it is possible that the HWA system is experienced differently in other regions. It is likely that the five municipalities in this research are representative of small to medium size municipalities. Future research could repeat the study in other neighborhoods to study similarities and differences between different neighborhood types in the same municipality.

## Conclusions

Inclusive qualitative research about HWAs from a systems perspective among citizens living in low SEP neighborhoods has yielded insights into their perceived LPTs. The extent to which HWA facilities and activities were utilized was influenced by ten overarching themes, of which social cohesion and familiarity had a prominent role, as also recognized by the co-researchers. LPTs indicated the combination of overarching themes needed to increase activity or facility utilization. Engaging low SEP citizens and conducting research with citizens as co-researchers may be a useful way in which to tailor HWA activities and facilities more to citizens’ needs.

### Electronic supplementary material

Below is the link to the electronic supplementary material.

Supplementary Material 1

Supplementary Material 2

## Data Availability

The data that support the findings of this study are available from Radboudumc but restrictions apply to the availability of these data, which were used under license for the current study and so are not publicly available. Data are, however, available from the authors upon reasonable request and with permission of Radboudumc. Therefore, data may be requested by emailing the quality team of our department of primary care (kwaliteitsteam.elg@radboudumc.nl).

## Footnotes

1 The topics are: healthy living description, personal circumstances, foot and cycle paths, sports facilities, playgrounds, green spaces, museums and theaters, community center, churches, healthcare, school, food supplies, contact with neighborhood, activities lacking, reaching citizens, and all activities as a separate topic.

## Abbreviations

SEP: Socioeconomic position
e.g: For example
HWA: Healthy weight approach
i.e: In other words / that is to say
LPT: Leverage point theme
WMO: Wet Medisch-wetenschappelijk Onderzoek met mensen (the Dutch Medical Research with Human Subjects Law)
E: Events
S: Structures
G: Goals
B: Beliefs
