# Supplementary material for: Citizens’ perspectives on healthy weight approaches in low SEP neighborhoods: a qualitative study from a systems perspective
Source: BMC Public Health. 2024 Aug 7;24:2137. doi: 10.1186/s12889-024-19595-3 (PMC11304654; doi:10.1186/s12889-024-19595-3)
Supplement: Supplementary file 1 — Supplementary Material 1 [file 12889_2024_19595_MOESM1_ESM.pdf]

Supplementary material 1

Interview protocol [translated from Dutch to English]

Hello, thank you for participating in this research. My name is [name] and I am going to talk to you today about nutrition and exercise in [name of neighborhood]. Before we start, I'd like to discuss some points with you.

- Can you hear and see me well?
- Do you prefer me to say You or you?
- We would like to know more about your experiences in the neighborhood. So, there are no right or wrong answers.
- The conversation will last about 30 minutes. The conversation may take 5 to 10 additional minutes because of reading the informed consent form out loud.
- Have you read the informed consent? It says that we would like to record this conversation. Is it okay if I start the audio recording now?

[Start recording]

Then we first want to go through the informed consent with you. Taking part in this research means that you:

- Have a conversation together with the researchers.
- It is okay for you that the conversations are being recorded. This way we can listen back to the conversation and write down what was said.
- No one except for the researchers know who you are.
- You can stop during the conversation. You do not have to say why you are stopping.
- If you don't want to answer something, you don't have to.
- We keep the information and documents on Radboudumc computers in a safe place for 15 years. Only the research team can open the documents.
- We make a report and presentation of the research, this does not contain your name.
- Everything you say will be known only by the researchers.

With permission you let us know:

- That you have received all the information about the research.
- All your questions have been answered.
- You agree with the agreements on the informed consent form.
- You want to participate in the research and therefore in a conversation about things in your neighborhood that have to do with a healthy lifestyle.

Normally we sign the form, but because the conversation goes through the computer, we record our name as signature.

Today [day, month, year], the researcher [own name] is conducting this interview.

Do you agree with the points just mentioned? Then give your first and last name as a signature.

Do you also give permission to use the information from this study for future research about a healthy lifestyle in your neighborhood?

Do you give permission to contact you again for follow-up research?

[Stop recording, start recording again]

Then we will start with the conversation now.

2.1 Individual conversation

1. What do you think about when you think of healthy living? [no follow-up questions]

2. Are there things you do to live a healthy life? If so, what is that? If not, why not? [no follow-up questions]

We are going to talk about your neighborhood. By neighborhood, we mean [name of neighborhood].

3. What are places in your neighborhood that you often visit?

Goal: to discover how we can reach citizens. Concrete places where we can stand and or put posters.

Possible follow-up questions: Where in … [name municipality/ mentioned place] is this?

4. Where in your neighborhood do you see other people walking or exercising? [no follow-up questions]

2.2 Facilities

Initially, the focus is always on the neighborhood. If the facility is not present in the neighborhood, ask whether they use the facility elsewhere in the municipality.

Example question: Do you use this [facility] elsewhere in [name municipality]?

Goal: What facilities are used? And which not? Why are they (not) used (so what is good and bad about them)? We are looking for things that we can change within our project group/this municipality. This can also be something new.

Use the sub-questions as questions for help and check them off during the conversation when they have been addressed.

5. What do you think about the cycle and foot paths in [name of neighborhood]?

- ☐ Which cycle and foot paths do you use?

- Can everyone use the cycle and foot paths? For example, think about safety or maintenance.
    - Why/why not? [if this is not clear from the previous answer]
- Are there things that are good or bad about cycle and foot paths?
- Is there anything else?

6. What do you think about the sports facilities in [name of neighborhood]?

- Which sports facilities do you use in your neighborhood? Would you use sports facilities if they were there?
- Can everyone use these sports facilities? For example, think about affordability or quality.
    - Why/why not? [if this is not clear from the previous answer]
- Are there any good or bad things about the sports facilities in [name of neighborhood]?
- Is there anything else?

7. What do you think about the recreation places in [name of neighborhood]? Think for example about playgrounds, green spaces, museums, theaters.

- Which recreational places in your neighborhood do you use?
- Can everyone use those places? For example, think about safety or maintenance.
    - Why/why not? [if this is not clear from the previous answer]
- Are there any good or bad things about the recreational places in [name of neighborhood]?
- Is there anything else?

8. What do you think about the facilities in [name of neighborhood]? For example, think of community centers, church, care, schools.

- Which facilities in [name of neighborhood] do you use?
- Can everyone use these facilities? For example, think of safety or maintenance.
  - Why/why not? [if this is not clear from the previous answer]
- Are there any good or bad things about the facilities in [name of neighborhood]?
- Is there anything else?

9. What do you think about the food offer, such as fruit and vegetables, restaurants, cafes, snack bars in [name of neighborhood]?

- What food offer do you use in your neighborhood?
- Can everyone make use of the food offer? For example, think about affordability or quality.
  - Why/why not? [if this is not clear from the previous answer]
- Are there any good or bad things about the food offer in [name of neighborhood]?
- Is there anything else?

10. How would you describe the contact with the neighborhood?

- Do you think it is important that you know your neighbors?
- In which places do you meet your neighbors?
- Is there anything else?

11. What other facilities help for a healthy lifestyle, healthy weight, exercise, and nutrition?

Goal: To find out whether there are other facilities that the citizen uses/deems relevant. We do not ask for all facilities.

12. We have talked about several facilities. Which facilities are most important to you when it comes to healthy lifestyle, healthy weight, exercise, and nutrition?

Possible follow-up question: which facilities do you use the most? What facility do you find lacking the most in your neighborhood?

2.3 List of activities

A lot is happening in your municipality in the field of healthy lifestyle, healthy weight, exercise, and nutrition. Here, you see a list of activities that take place in your municipality. We will go through them 1-by-1.

[Put A4 on the table]

Goal: Which activities are (not) known? Which activities are (not) used? Why are they or are they not used (so what is or is not good about them)? We are looking for things that we can change within our project group/the municipality. This can also be something new. **Follow-up questions are below the numbers.**

13. Are you familiar with [name activity]?

- [If yes] -a. Do you use [name of activity]?

  If other target group (children, elderly): Do you know people who use [name of activity]?

  - [If yes] b. What do you like or dislike about [name of activity]?

    What do they think is good or bad about [name of activity]?

  - [If no] b. Why not?

A list of activities per municipality is provided:

- **[Name activity]** (target group if only for children or elderly) – short explanation of the activity if the name itself is unclear
- **...**

14.

a. How could we give attention to these activities?

b. Where do you really respond to??

Goal: Much is being done to promote these activities from different organizations and through different ways, but it is often unfamiliar. How can we increase awareness? By what means can this be done (such as social media, newspaper, poster)? What does this look like practically? What kind of text or content would appeal?

Possible follow-up question: What do you mean by that? How could we do this?

15.

a. What activities for a healthy weight do you find to be lacking?

b. What would help you to maintain a healthy weight?

Goal: Looking for ideas for new activities. What would help the citizen? Where is the need? What do citizens encounter?

2.4 Background data

16. Do you ever think about losing weight?

May I ask: how much do you weigh and what is your height?

** If you think that the answer is incorrect, write this down as an observation (provide an estimation of how much the weight should be higher or lower)*

17.

a. Which education or school did you finish??

b. What level of education is this? [Only if you don’t know / have doubts]

18. Gender [*check yourself, don’t ask*]

- ☐ Male

- Female

19. Age ***[check yourself, don't ask]***

- Youth (15–18)
- Young adults (18–35)
- Adults 35–65
- Elderly (65+)

2.5 End of interview

These were all the questions in the conversation. Thank you for your participation in the interview!

[Check yourself]

20. Would you like to receive the report with the research results? We can send this to you by email, it will take a few months.

- Interested
- Not interested

We would like to thank you for your participation with a Hema gift card of €7,50. Are you interested in this?

21. Interest in HEMA gift voucher

- Interested
- Not interested

Then we will send it to your email address, please also check your spam mailbox.

Do you have any further questions?

Then I wish you a nice day!

[Citizen leaves Teams – continue recording]

- Record gender + age category.
- If you think weight and height are incorrect, please mention this as well.

[stop recording]
