# Supplementary material for: Citizens’ perspectives on healthy weight approaches in low SEP neighborhoods: a qualitative study from a systems perspective
Source: BMC Public Health. 2024 Aug 7;24:2137. doi: 10.1186/s12889-024-19595-3 (PMC11304654; doi:10.1186/s12889-024-19595-3)
Supplement: Supplementary file 2 — Supplementary Material 2 [file 12889_2024_19595_MOESM2_ESM.pdf]

Supplementary material 2

Usage of the Action Scale Model

ASM-level (Nobles et al., 2021)	Definition (Nobles et al., 2021)	Operationalisation	Example
**1. Events**	Action is mainly focused on "observable behaviours and outcomes of stakeholders within the system (e.g. symptoms of the system)".	Mainly includes citizens' behaviours and outcomes regarding facilities and activities in the neighborhood, such as mentioning that an activity or facility exists, participating in activities or using facilities.	Some citizens used the community centre, while others did not.
**2. Structures**	Action is mainly focused on "the organization of the system that causes these events to occur (e.g. physical structures, patterns, relations, information streams)".	Mainly includes the structures that facilitate or discourage citizens to (not) participate in activities or (not) use facilities, such as physical structures, appreciation of activities and facilities, information streams.	Most citizens were familiar with the community centre, and perceived the community centre as a meeting place were for instance Dutch lessons were organized.
**3. Goals**	Action is mainly focused on "the ambitions the systems work towards".	Mainly includes citizens' goals to (not) participate in activities or (not) use facilities. Goals explain where citizens live towards.	A citizen aims to have extra Dutch lessons and get in contact with other people.
**4. Beliefs**	Action is mainly focused on "deeply held norms, attitudes and values about elements of the system".	Mainly includes the underlying reasons, such as norms, attitudes and values. Beliefs explain why a person perceives the goals as important.	A citizen felt excluded from the community centre.
